# Overexpression of GSN could decrease inflammation and apoptosis in EAE and may enhance vitamin D therapy on EAE/MS

**DOI:** 10.1038/s41598-017-00684-w

**Published:** 2017-04-04

**Authors:** Jifang Gao, Zhaoyu Qin, Xinyuan Guan, Juanjuan Guo, Huaqing Wang, Shilian Liu

**Affiliations:** 1 Department of Biochemistry and Molecular Biology, School of Medicine, Shandong University, 44#Wenhua Xi Road, Jinan, 250012 Shandong P.R. China; 2 Laboratory of Systems Biology, Institute of Biomedical Sciences, Fudan University, 138# Medical School Road, Shanghai, 200032 China; 3 Shouguang Century School, 789#Nongsheng Street, Shouguang, 262700 Shandong China

## Abstract

The decrease of gelsolin (GSN) in the blood has been reported in multiple sclerosis (MS) patients and experimental allergic encephalomyelitis (EAE) animals, but the protective effect of GSN on EAE/MS lacks of evidence. In our study, we increased the GSN level in EAE by injecting GSN-overexpress lentivirus (LV-GSN) into the lateral ventricle and caudal vein and found that GSN administration can delay the onset and decrease the severity of EAE. Vitamin D is proven to have a therapeutic effect on MS/EAE; however, we previously found that vitamin D caused a downregulation of GSN, which might limit vitamin D efficacy. In our current research, we obtained a better symptom and a slowing down progression in EAE after combining vitamin D treatment with a proper increase of GSN. Furthermore, we discovered that the mediation of vitamin D on GSN might occur through the vitamin D receptor (VDR) by using gene interruption and overexpression to regulate the level of VDR in PC12 cells (a rat sympathetic nerve cell line). We also confirmed the anti-apoptotic function of GSN by GSN RNA interference in PC12. Collectively, these results support the therapeutic effect of GSN in EAE, which might enhance Vitamin D therapy in EAE/MS.

## Introduction

Gelsolin is the most widely expressed actin-severing protein in humans^1^; not only is it mainly expressed in muscle tissue^2^, but it is also present in the nervous system, including myelin-forming cells^3^, neurons^4^, and choroid plexus^5^. It exists as 2 isoforms in the human body, a cytoplasmic and a secreted isoform, which are structurally similar but not identical. Both forms are encoded by a single gene located on chromosome 9 in humans^6–8^, but the secreted form (plasma gelsolin, pGSN) differs from the intracellular form (cytoplasmic gelsolin, cGSN) by an additional unique leader peptide (24 amino acids) at its N-terminal^9^. The most extensively examined role of pGSN is scavenging extracellular actin filaments released from dead cells into the blood^10^. In addition, pGSN can also bind to proinflammatory and bioactive molecules, including sphingosine 1-phosphate (S1P)^11^, lipopolysaccharide^12^, and lysophosphatidic acid^13, 14^, which could temper host inflammatory responses during certain disease progression. In various organ injuries and neurological conditions, including multiple sclerosis (MS), GSN concentrations in cerebrospinal fluid (CSF)^15^ and the blood^16^ decrease significantly. It has been reported that the injection of the recombinant gelsolin into animal models in appropriate doses can decrease mortality and reduce the damage due to hyperoxia, burn, and sepsis conditions^17–19^. cGSN expression could be induced during inflammatory processes, but the role cGSN plays in the subsequent inflammation-induced neural apoptosis is crucial^20^. It is reported that the full-length gelsolin and its C-terminal half mostly have an anti-apoptotic function; in contrast, the N-terminal half of gelsolin potentially plays a pro-apoptotic role^21^; however, the relationship between cGSN and MS has not yet been reported. Our previous research first suggested that cGSN might play a protective role against apoptosis in the MS process^22^.

MS and its rat model experimental autoimmune encephalomyelitis (EAE) are considered to be the most common inflammatory-mediated autoimmune demyelinating disorder of the central nervous system (CNS) of unknown etiology^23^, consisting of an inflammatory response, autoimmune reactivity, and nerve cell apoptosis processes^24, 25^. Recently diagnostic imaging of neurological disorders and therapy options for MS patients have been greatly improved^26, 27^, but a complete understanding of the pathology and the available definitive cure for this complex disease is still regrettable. The inflammatory response in MS and EAE is partly induced by the infiltration of anti-myelin CD4+ T lymphocytes into the brain, and subsequently, the CD4+ T lymphocytes in the brain could lead reactive astrogliosis to synthesize proinflammatory cytokines to CNS^28, 29^. Neuron and oligodendrocyte (ODC) apoptosis are demonstrated during MS and EAE^30–32^. The ODCs are myelin and the myelin-producing cells, and their loss is directly associated with neuronal dysfunction and damage leading to the clinical manifestations of the disease^32^.

Vitamin D is well known for its calcium homeostasis regulating function; however, in recent years, the study of vitamin D began to shift to immune system diseases^33^. An epidemiological survey has found that there is a close correlation between the incidence of MS and light^34^, and since light is the catalyst of vitamin D synthesis in body, people presume that light affects the levels of vitamin D and thus, affects the incidence of MS^35^. Subsequent experiments present that 25- (OH)_2_D_3_ level in MS patients is significantly lower than normal^36, 37^, and animal experiments in EAE prove that dietary supplement of vitamin D during the acute stage of EAE can effectively delay the onset time and alleviate the symptoms, but cannot prevent the development of EAE completely^38^ (our previous animal experiments also got the same results^39, 22^).

The biologically active form *in vivo* of vitamin D_3_ is 1,25- dihydroxy vitamin D_3_(1a, 25(OH)_2_D_3_), which initiates its biological responses by binding to the vitamin D receptor (VDR). VDR is a kind of a DNA-binding transcription factor that is widely distributed in humans^40^. After combining with 1a, 25(OH)_2_D_3_, VDR forms a heterodimer with retinoid X receptor (RXR), then the heterodimer binds specific genomic sequences (vitamin D response elements, or VDREs) following acting to influence gene transcription^41^. Besides VDRE, VDR also could control gene transcription by associating with a number of transcriptional co-regulators^42^. In recent years, a lot of genes regulated by VDR have been identified due to combining chromatin immunoprecipitation assays with sequencing (ChIP-seq) technology^41, 43–45^; these genes are associated with diseases, such as systemic lupus erythematosus (SLE), rheumatoid arthritis, chronic lymphocytic leukemia, colorectal cancer, and multiple sclerosis^41^.

In our previously study, we have found that the therapeutic effect of vitamin D on EAE is derived from its ability to reduce S1P, but on the other hand is limited by its simultaneous effect in reducing pGSN and cGSN^22^. In the current research, we sought to examine the therapeutic effect of GSN on EAE and the relationship between GSN and vitamin D, and finally, to find out whether overexpressed GSN could enhance vitamin D therapy in EAE. By the injection of GSN overexpression lentivirus (LV-GSN) into the ventricle and caudal vein, we observed a significant remission of EAE, meanwhile, a better effect was found when the vitamin D supplement combined with GSN overexpression rather than individually. We also discovered that vitamin D could affect the concentration of GSN through VDR by using shRNA lentivirus interrupting VDR expression.

## Results

### LV-GSN Administration Decreased Disease Severity of EAE

**Figure 1:**
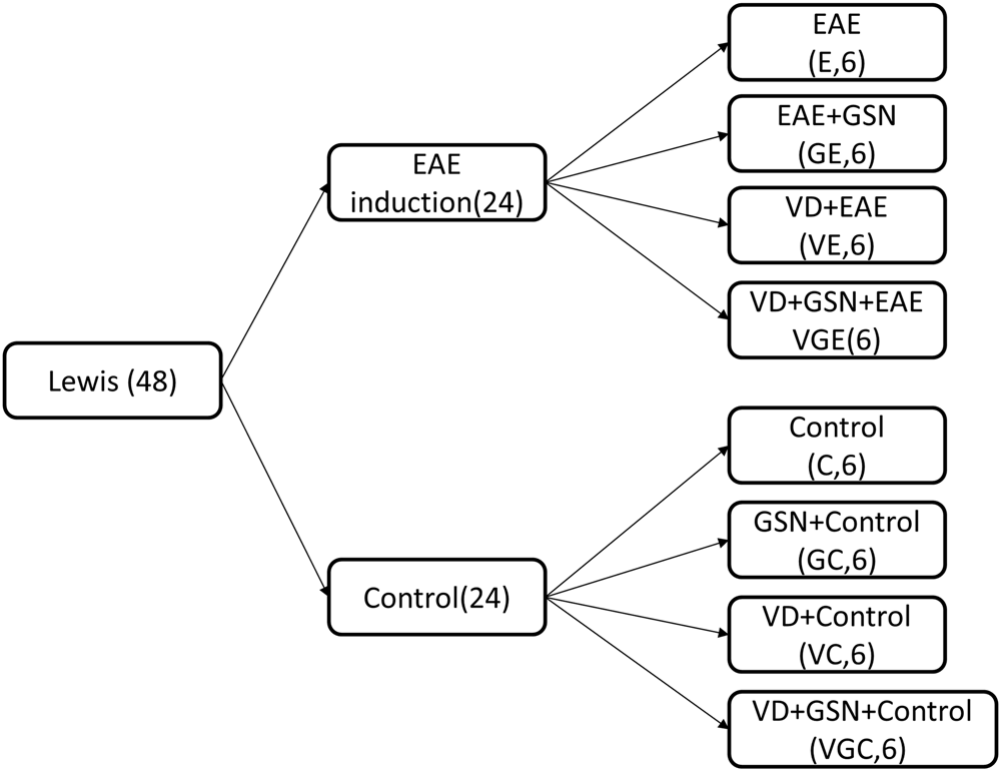
48 Lewis male rats were first divided into 2 groups based on EAE induction (EAE) or not (control). Then each group was divided into 4 groups. The first group was treated with vitamin D_3_ (VC and VD), the second group was injected with LV-GSN (GC and GE), the third group was both treated with vitamin D_3_ and injected with LV-GSN (VGC and VGE), and the last group was without treatment (C and E).

**Figure 2:**
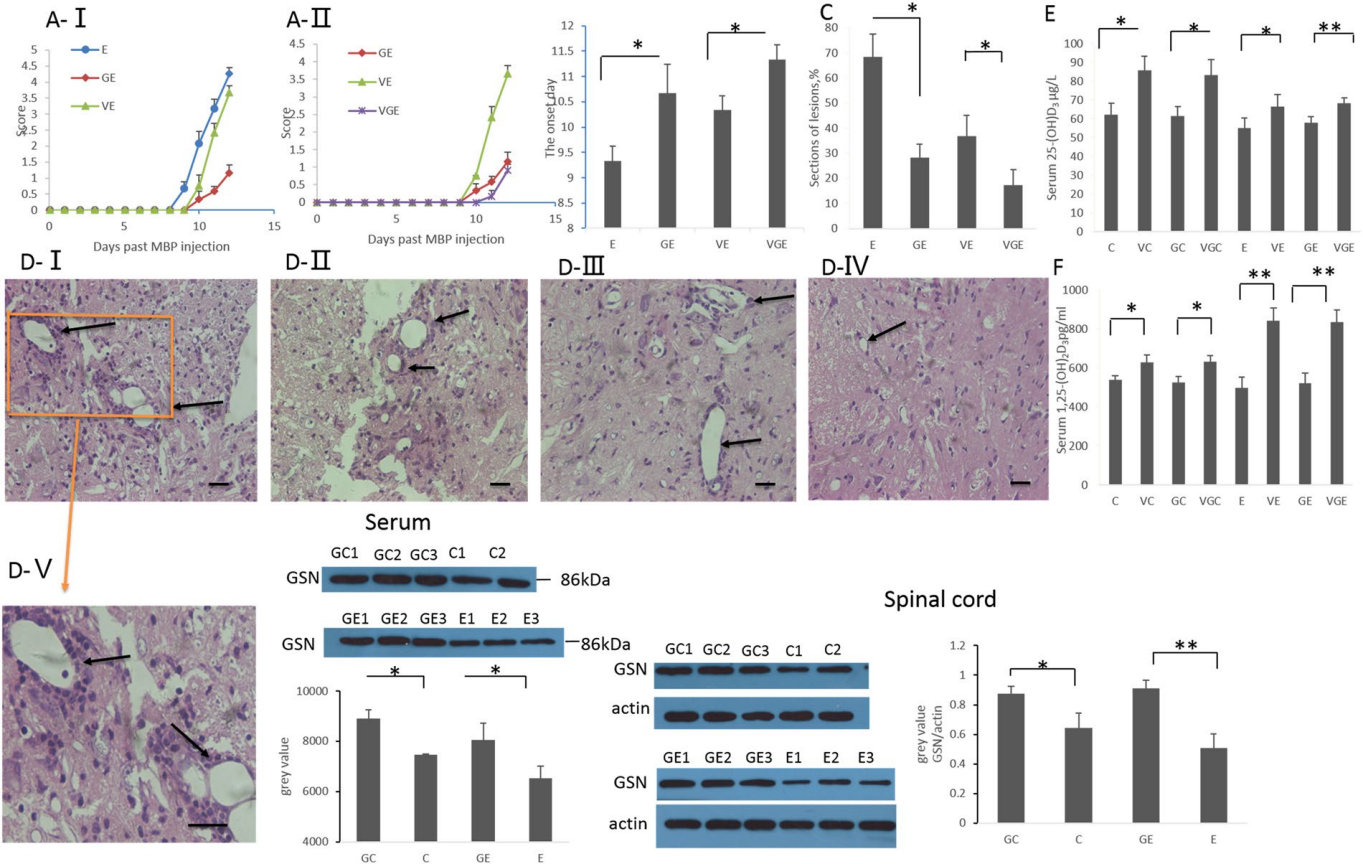
LV-GSN and vitamin D_3_ supplementation delays the onset of EAE and reduces inflammation. (**A**) Effects of LV-GSN, vitamin D_3_ and the combination of them on the course of EAE disease progression as evaluated by severity scoring. *p < 0.05. Shown is the composite mean from 17–18 mice/group evaluated in three separate experiments. (**B**) Average time of onset of EAE for rats fed with (VE) and without (E) vitamin D, injected with LV-GSN (GE), and injected with LV-GSN during vitamin D supplement (VGE) is shown. *p < 0.05. Shown is the composite mean from 17–18 mice/group evaluated in three separate experiments. (**C**) Statistical analysis of lesions in the spinal cord of E, GE, VE, and VGE rats. *p < 0.05. (**D**) Histopathological EAE disease in the spinal cord of E (D-I), VE (D-II), GE rats (D-III) and VGE rats (D-IV); D-Vis the higher magnification image of the marked part of D-I (representative images from 3 rats per group). The inflammatory lesions are indicated by arrows. (D-I, D-II, D-III, D-IV, ×200 magnification; D-V, ×400 magnification, scale bar 10 μm). (**E**) The concentration of 25-(OH) D_3_ in serum was measured by ELISA for rats fed without and with vitamin D supplementation in the control (C and VC), acute stage EAE (E and VE), and accompanied by LV-GSN (GE and VGE, GC, and VGC) groups. **p < 0.01, *p < 0.05. (**F**) ELISA was performed to measure the concentration of 1,25-(OH)_2_D_3_ in the serum of the same groups of rats as in panel E. **p < 0.01, *p < 0.05. (**G**) Western blot and real-time PCR was performed to detect the efficiency of LV-GSN in increasing the expression of GSN in serum. (**H**) The change of GSN level (protein and mRNA) in the spinal cord before (**E**) and after (GE) LV-GSN injection, *p < 0.05 Data are mean ± SD. **p < 0.01, *p < 0.05 (one-way ANOVA). The blots shown in Fig. 2G-I and 2H-I are cropped; the uncropped full-length gels are presented in the Supplementary Figure (Fig. S1C,D).

Our previous research proved that increasing the GSN level in rats might have a therapeutic effect on EAE^22^. To determine the effects of GSN in the animal model, we established the EAE model in our laboratory. 48 rats used in this research were divided into 8 groups (group E, GE, VE, VGE, C, GC, VC, VGC) based on different treatments, the details of the groups are presented in Fig. 1. Rats were injected with GSN-overexpressed lentivirus (LV-GSN) or control lentivirus into the lateral ventricle and vena caudalis, and the concentrations of GSN in the spinal cord and serum were measured by Western blotting to ensure that LV-GSN could cause a significant increase in GSN. The levels of GSN expression in rats with GSN injection (groups GE and GC) are apparently higher than that in rats with the control lentivirus injection (groups E and C), no matter if EAE was induced or not. The results are shown in Fig. 2G and H, which indicate the LV-GSN could successfully increase the level of GSN expression in rats.

To ascertain the effects of GSN on the development of EAE, we calculated disparities in the onset of the disease. The first sign of EAE appeared at about Day 9 after EAE induction immunization in rats following no other treatment (group E), but it appeared at about Day 10.5 in rats immunized with MBP and injected with LV-GSN (group GE). There was about a 1.5 day delay between group E and group GE rats (Fig. 2B), thus verifying a protective effect of GSN in the EAE model. We also calculated the severity score following EAE induction for 12 days to determine the long-term effects of GSN treatment. The results showed that group GE rats had a delayed start of the disease and decreased inflammation and the highest severity score in the GE rats was obviously lower than the E rats (Fig. 2A–I). Furthermore, the H&E stained paraffin sections of the spinal cords showed that the levels of inflammation and accumulation of infiltrated cells were reduced in the spinal cord of LV-GSN injected rats (Fig. 2D), which was verified statistically (Fig. 2C). These results suggested that LV-GSN administration can delay the onset of EAE and decrease disease severity of EAE.

### LV-GSN Administration Could Enhance Vitamin D Therapy in EAE

Vitamin D deficiency is associated with an increase in the risk of developing MS^20, 21^. In our former study we demonstrated that vitamin D could delay the onset of EAE, but could not prevent disease, and this limitation might be associated with the decrease of GSN by vitamin D^22^. In order to determine whether a combined therapy of GSN and vitamin D might produce a more beneficial effect in treating MS/EAE, we treated rats with or without vitamin D supplementation during LV-GSN or control lentivirus injection. To ensure that vitamin D was effectively absorbed by the rats, we performed ELISA to measure the concentrations of 25-(OH) D_3_ and 1,25-(OH)_2_D_3_ in the serum. The results showed that in serum the concentrations of 25-(OH) D_3_ and 1,25-(OH)_2_D_3_ are higher in all of the groups of rats that had vitamin D supplementation (groups VC, VGC, VE, and VGE) than that in rats without vitamin D supplementation (groups C, GC, E, and GE) (Fig. 2E and F). Therefore, these results imply that additional dietary vitamin D_3_ is effectively absorbed by all 4 groups of rats.

The results of calculating differences in the onset of the disease show that the first sign of EAE appeared at about Day 10 in EAE rats treated with vitamin D (group VE). In EAE rats injected with LV-GSN during treatment with vitamin D (group VGE), the first sign of EAE appeared at about Day 11.5 and there was a delay of about 1 day compared with rats treated with vitamin D (group VE) or injected with LV-GSN only (group GE) (Fig. 2B). These results verify a better effect of combined GSN with vitamin D in the EAE model. The 2 weeks used to calculate the severity score presents that VGE rats had a delayed start of the disease and lower severity score compared with GE and VE rats (Fig. 2A-II,2B). Furthermore, the results of H&E staining show a lower level of inflammation and accumulation of infiltrated cells in the spinal cord of VGE rats (Fig. 2D). These results suggest that LV-GSN administration combined with vitamin D supplementation could enhance vitamin D therapy in EAE.

### Levels of pGSN Decreased in MS Patients

**Figure 3:**
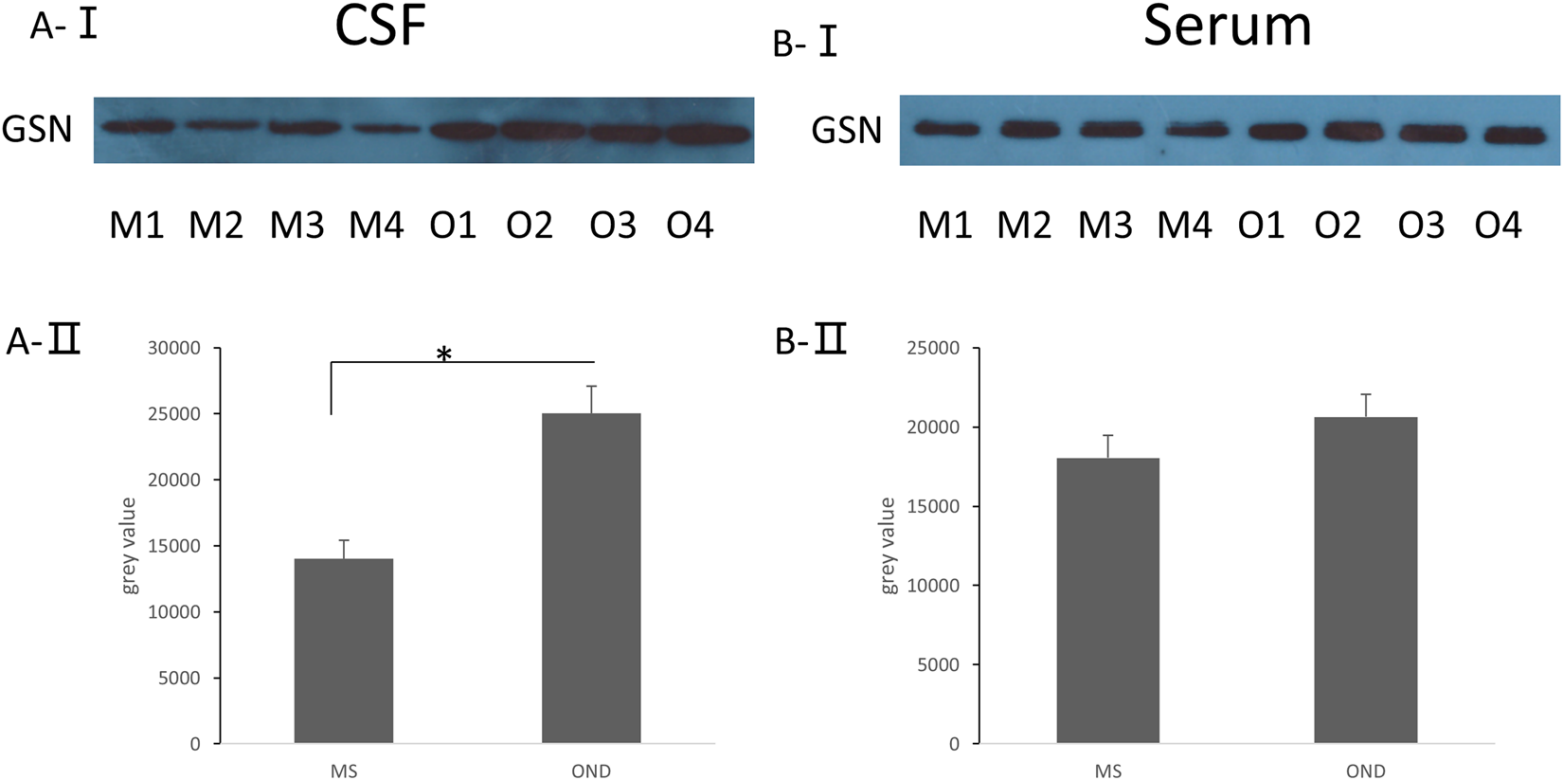
The expression of pGSN is decreased in the CSF, but only changed slightly in the serum of MS patients. **A-I**, Analysis of pGSN levels in the CSF of MS patients (M1-M4) or OND patients (O1-O4) by Western blot. **A-II**, We calculated the relative volume percentage of each band using Quantity One software. *p < 0.05 (one-way ANOVA). **B-I**, Analysis of pGSN levels in the serum of MS patients (M1-M4) or OND patients (O1-O4) by Western blot. **B-II**, We calculated the relative volume percentage of each band using Quantity One software.

In our previous proteomic studies, we used 2D-DIGE technology to screen the proteins which were expressed differently in the CSF proteome of MS and OND patients. The results suggest that pGSN is more than 1.5-fold downregulated in the CSF of MS patients^39, 46^. In order to confirm the findings, we detected the concentration of pGSN in the CSF and serum from 4 MS and OND patients. The results present that the levels of pGSN in MS patients are verified to be nearly 3-fold reduced in CSF (Fig. 3A), and slightly decreased in serum (Fig. 3B). The same findings were already demonstrated in our previous study^22^. The differential expression of pGSN in the serum and CSF of MS and OND patients indicate that the expression of GSN could correlate with the MS state.

### VDR was Upregulated after Vitamin D Supplementation while Both pGSN and cGSN were Downregulated

**Figure 4:**
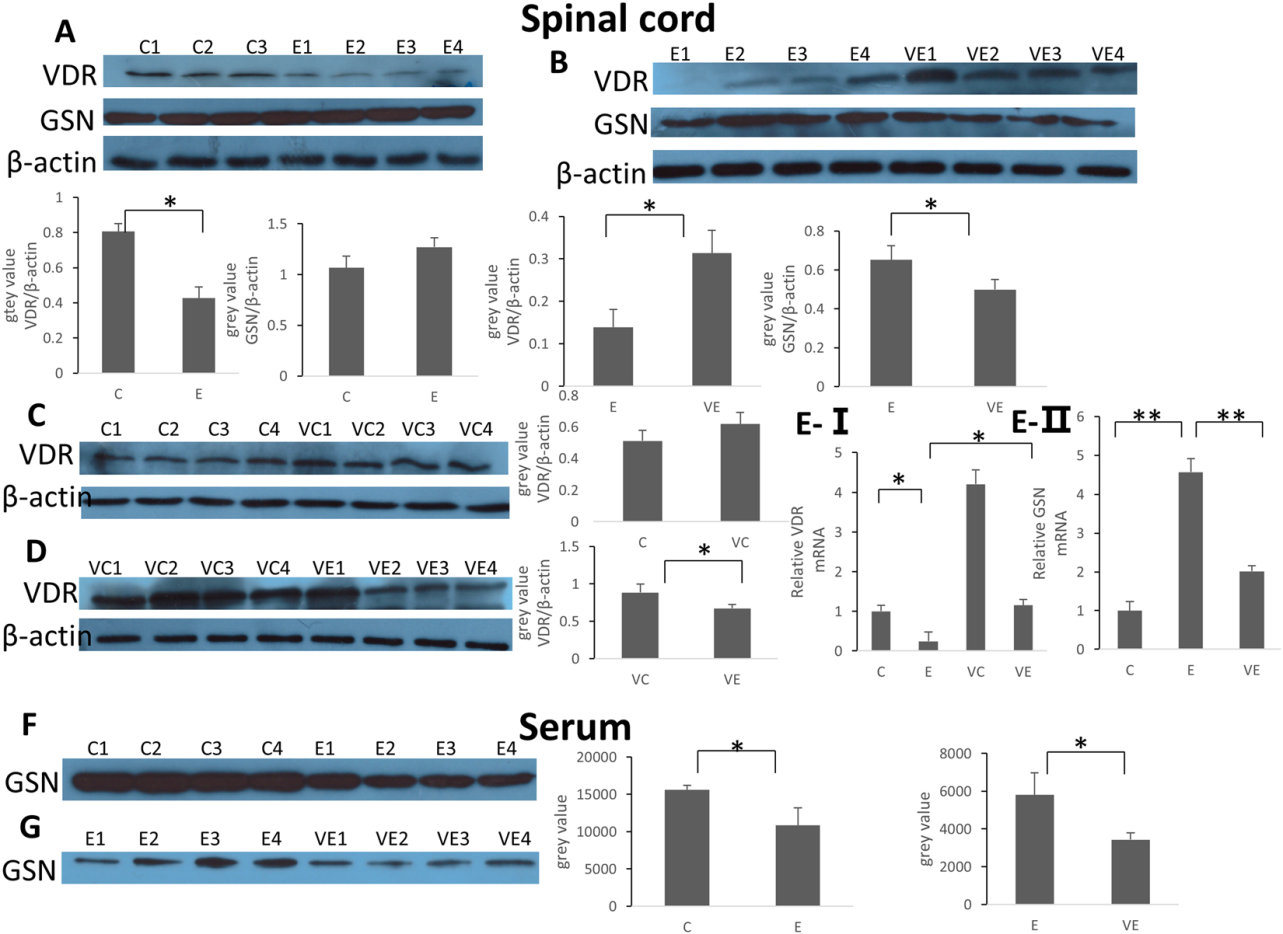
The changes of VDR and GSN levels after EAE induction or vitamin D supplementation. (**A**) Western blotting of VDR and cGSN in the spinal cord of the control and EAE rat groups. (**B**) Western blotting was performed to compare the levels of VDR and cGSN in the spinal cords of group E rats (E1–4) to the levels in group VE rats (VE1–4). (**C**) Western blotting of VDR in the spinal cords of the control rats without (C1–C4) with (VC1–VC4) vitamin D supplementation. (**D**) Western blotting of VDR in the spinal cords of rats with vitamin D supplementation with or without EAE induction. (**E**) Real-time PCR of VDR or GSN mRNA levels in the spinal cords of rats from each group. Results (means + SD of triplicates) are representative of 3 independent experiments. (**F**) Western blotting analysis of the levels of pGSN in the serum of the control rats (C1–4) compared to the levels in the EAE rats (E1–4). We calculated the relative volume percentage of each band using Quantity One software. *p < 0.05 (one-way ANOVA). (**G**) Western blotting comparison of pGSN in the serum of EAE rats with EAE (E1–4) fed vitamin D supplements (VE1–4). We calculated the relative volume percentage of each band using Quantity One software. *p < 0.05 (one-way ANOVA).

Considering that the function of vitamin D *in vivo* is due to the vitamin D receptor (VDR), we detected whether VDR might be modulated by vitamin D in EAE. The results show that, in spinal cords, VDR levels are significantly lower in EAE (groups E and VE) rats than the control rats (groups C and VC) (Fig. 4A,B). When the comparison comes to the groups with or without vitamin D supplementation, the levels of VDR are increased after the vitamin D supplement (groups VC and VE) (Fig. 4B,C). Western blotting results were verified at the mRNA level using real-time PCR (Fig. 4E–I). These results suggest that the level of VDR in rats may have some relationship with EAE development, and it could be regulated by vitamin D.

We previously found out that pGSN decreased during EAE, and in this study, we carried out Western blots to reconfirm whether GSN might also be regulated in EAE by vitamin D. The results are in line with what we already reported^22^. As is shown in the results, we observed a marked decrease of the pGSN level in serum in EAE rats compared with the control rats (Fig. 4F), but in the spinal cords, cGSN was observed at a similar level or was increased slightly in EAE rats (Fig. 4A). Furthermore, we also compared the GSN levels between the groups with and without vitamin D supplementation, and the results show that vitamin D causes a reduction of both pGSN in serum (Fig. 4G) and cGSN (Fig. 4B) in the spinal cords. Western blotting results were verified at the mRNA level by real-time PCR (Fig. 4E-II). Collectively, these results indicate that during the induction of EAE, pGSN in the serum is decreased while cGSN in the spinal cord is not changed; however, vitamin D could cause a decrease in both pGSN and cGSN.

### Vitamin D Regulated the Expression of GSN due to its Binding of VDR

**Figure 5:**
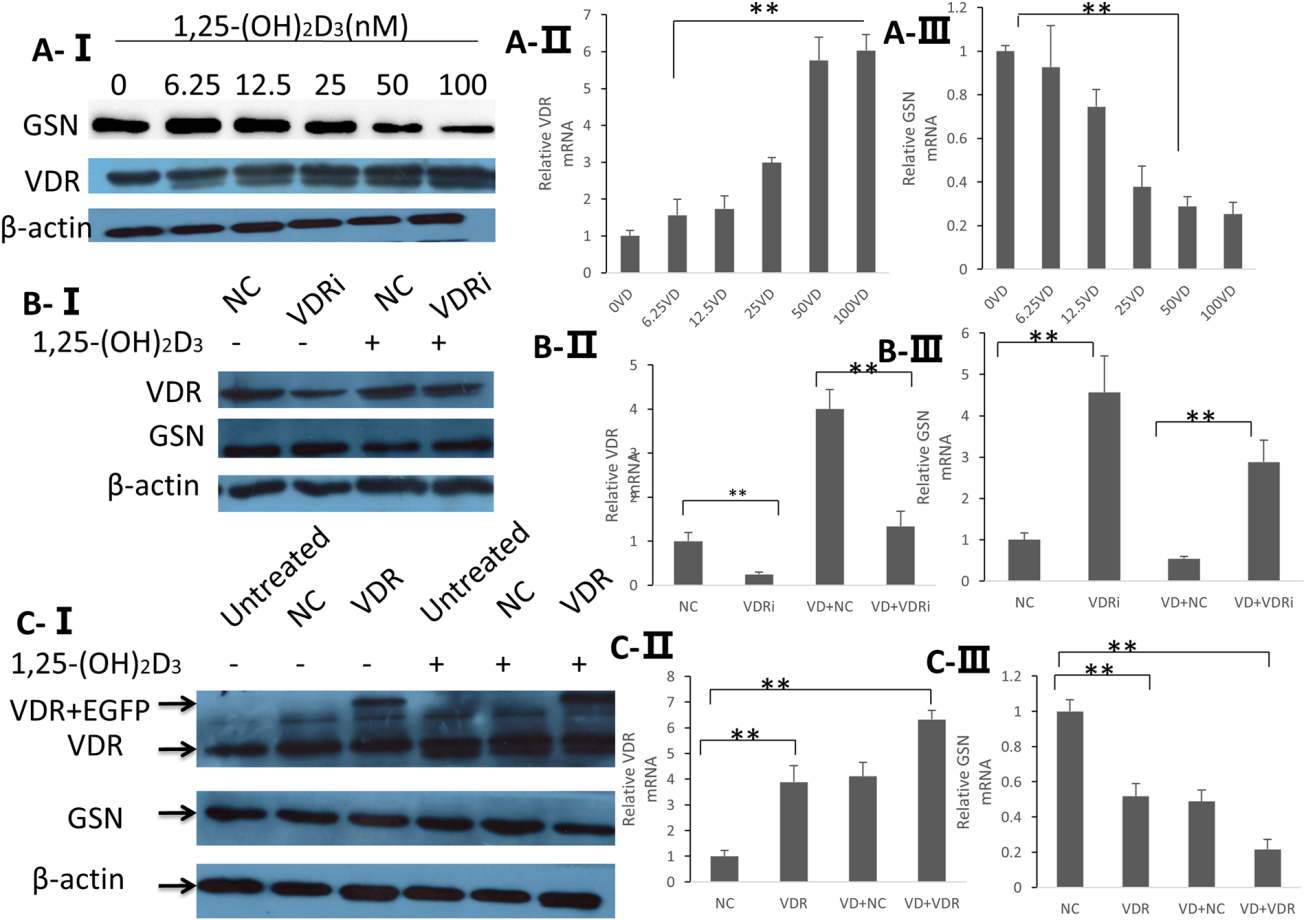
**A-I**, GSN and VDR expression in the PC12 cells was verified by Western blot analysis following stimulation by 1,25-(OH)_2_D_3_ (0, 6.25, 12.5 25, 50, and 100 nmol/L) for 72 h. **A-II, A-III** the mRNA levels were examined by real-time PCR. **B**, PC12 cells stimulated for 24 h with or without 1,25-(OH)_2_D_3_ (50 nmol/L) following transfection with the VDR plasmid (GV230) or control (NC) plasmid. **B-I**, Western blot results of VDR and GSN levels, the blots are cropped; the uncropped full-length gels are presented in the Supplementary Figure (Fig. S1A). **B-II, B-III**, Real-time PCR tested the changes of VDR and GSN in the mRNA level. **C-I**, Western blot analysis of VDR interference in PC12 cells following injection of the VDR lentiviral expression vector (with EGFP gene) and control lentiviral expression vector. PC12 cells were stimulated with or without 1,25-(OH)_2_D_3_ (50 nmol/L) for 24 h before injection of VDR lentiviral expression vector and control lentiviral expression vector. **C-II, C-III**, Real-time PCR tested the changes of VDR and GSN in the mRNA level. *p < 0.05 (one-way ANOVA).

The bioactive form *in vivo* of vitamin D is 1,25-(OH)_2_D_3_. In order to detect whether vitamin D could regulate the levels of GSN and VDR *in vitro*, we stimulated PC12 cells with different concentrations of 1,25-(OH)_2_D_3_ (0, 6.25, 12.5, 25, 50, and 100 nmol/L) for 72 h. Western blot results show an obvious increase in the VDR level, but in contrast, the expression of GSN decreased significantly with the increase of 1,25-(OH)_2_D_3_ concentration (Fig. 5A–I). Furthermore, we performed VDR gene interruption and overexpression to find out whether the vitamin D mediation on GSN expression was through the vitamin D receptor (VDR). In order to increase the level of VDR in cells we transferred a VDR expression plasmid (GV230) into PC12 cells to facilitate the stable expression of VDR. PC12 cells were stimulated for 24 h with or without 1,25-(OH)_2_D_3_ (50 nmol/L) before being transfected with the VDR plasmid (GV230) or control plasmid (NC). At 48 h and 72 h after transfection, cells were collected for total RNA and protein extraction. Western blotting and RT-PCR were carried out to examine the efficiency of the VDR expression plasmid and its effect on GSN. Following Western blots, we observed that the levels of GSN were reduced in PC12 cells transferred with VDR plasmid in comparison with the NC plasmid, and the reduction was enhanced by stimulation with 1,25-(OH)_2_D_3_ (Fig. 5C–I). In the PC12 cells that were transferred with VDR interruption shRNA lentiviral expression vector (VDRi), the GSN levels were significantly increased while the VDR levels were downregulated by VDRi, and the increase of GSN by VDR interruption could be weakened by stimulation with 1,25-(OH)_2_D_3_ (Fig. 5B–I). The results of RT-PCR at the RNA level were similar to the Western blot results (Fig. 5A-II, B-II, C-II, A-III, B-III, C-III). These results support that the regulation of vitamin D on the expression of GSN relies on the binding of VDR.

### cGSN Mediated Apoptosis induced by TNF-α in EAE rats

**Figure 6:**
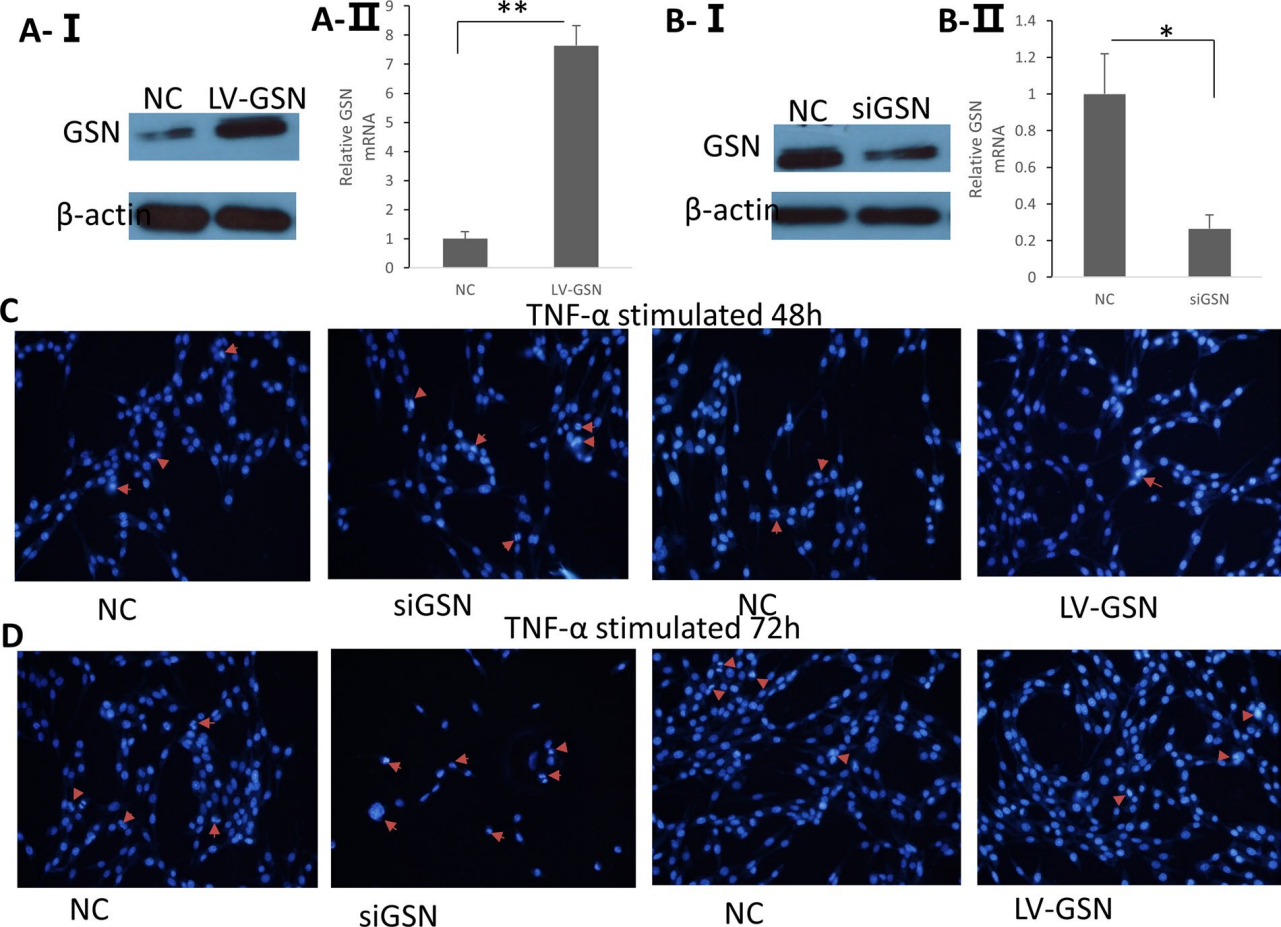
GSN expression has a deep relationship with decreased apoptosis. (**A**) GSN overexpression was verified by Western blot analysis and real-time PCR following transfection with LV-GSN or control (NC) lentivirus for 72 h. (**B**) GSN interference in PC12 cells was confirmed by Western blot analysis and real-time PCR following transfection with siGSN or control (NC) siRNA oligonucleotides for 72 h. The blots shown in Fig. 6A and B are cropped; the uncropped full-length gels are presented in the Supplementary Figure (Fig. S1B). (**C,D**) Levels of apoptosis were assayed by Hoechst 33258 staining 48 or 72 h following transfection and exposure to TNF-α. Images of the stained cells were captured under a fluorescent microscope at 350 nm stimulation and 460 nm emission (×100 magnification).

cGSN can be cleaved by caspase-3 upon activation of apoptosis^20^. In our former study, Western blot and flow cytometry were performed to assess whether the level of cGSN in EAE may have a relationship with apoptotic activity and the results supported a role for cGSN in inhibiting apoptosis^22^. To confirm the protective effect of cGSN from apoptosis, we used Hoechst 33258 staining to assess the differences of apoptosis in PC12 cells transferred with LV-GSN, control lentiviral, siGSN (siRNA against GSN), or control RNA (NC). The effectiveness of siGSN and LV-GSN in changing cGSN protein levels was examined at 72 h post-transfection by Western blot and RT-PCR (Fig. 6A,B). To induce apoptosis, the transferred cells were then treated with 20 ng/ml TNF-α for 48 h or 72 h. As shown in Fig. 6C,D, following transfection with siGSN, significantly more PC12 cells exhibited marginalization and nucleic chromatin condensation compared with the NC transfected cells. In contrast, transfection with the LV-GSN resulted in a decreased number of PC12 cells with nucleic chromatin condensation compared with the cells transfected with the NC inhibitor. These data verify that cGSN plays a role in inhibiting apoptosis.

Taken together, these results support that GSN plays a protective role in the EAE process, and the level of GSN could be mediated by vitamin D through VDR. Increased GSN levels *in vivo* during vitamin D treating MS/EAE will produce a more beneficial effect.

## Discussion

In this study, we found that while vitamin D and GSN could both reduce the symptoms of EAE rats, there was an unexpected decrease in GSN concentration during vitamin D therapy on EAE. For the first time we discovered that after increasing the GSN level during vitamin D treatment in EAE, better symptom reduction and a slowed down progression in EAE were obtained no matter whether compared with GSN or vitamin D supplement individually. Furthermore, we also found that the mediation of vitamin D on GSN level might occur through VDR.

Over the past few years, an increasingly more effective new agent for MS has been developed, but until now, a curative therapy that can both relieve the symptoms and slow the progression of the disease is still desired. Inflammatory response, autoimmune reactivity, and nerve cell apoptosis processes are all included during MS^24, 25^. There is growing interest in the connection between GSN and MS because of the potential neuroprotective properties of GSN. pGSN can scavenge pro-inflammatory signals, such as actin and lipopolysaccharidet, to ameliorate deleterious inflammatory response, while cGSN has been implicated in numerous biological processes, including cell motility, apoptosis, and phagocytosis^47^. In previous studies, we observed a > 1.5-fold decrease of pGSN in MS and OND patients by using 2-D DIGE^39, 46^, and in current research we confirmed the results by Western blotting. In EAE (the rat model of MS), a decrease of pGSN in the serum was also found in this study. These results suggest that pGSN might have a close relationship with the pathological process of MS. It has already been reported that recombinant human pGSN (rhp-GSN) can remit some inflammatory diseases in the animal model, such as burn injury, middle cerebral artery occlusion, and sepsis. Recently, Kevin Li-ChunHsieh *et al*. found that rhp-GSN could decrease myeloperoxidase activity and extracellular actin in the brain of EAE mice, resulting in less severe clinical disease and reduced disease activity^48^. This is highly consistent with the results we achieved in our current research. All of these results suggest that gelsolin might have a therapeutic effect on EAE, therefore, it could be a potential therapeutic target for MS.

Although the relationship between cGSN and MS has not been reported on much yet, the association of cGSN and apoptosis has been deeply researched. Full-length cGSN, and the C-terminal half of cGSN are generally considered as having anti-apoptotic function. Support for this notion comes from studies which showed that gelsolin overexpression could inhibit the loss of mitochondrial membrane potential and cytochrome C release from mitochondria, which finally inhibit activation of caspase-3, −8, and −9^49^. Previous studies have demonstrated that when PC12 cells were under oxidative stress the expression of cGSN was upregulated^50, 51^, which possibly was a response mechanism to neutralize the actions of apoptotic factors. Moreover, research in animal models have already been carried out and an *in vivo* study in liver failure mice showed that cGSN is increased during the disease process, and a significantly higher number of apoptotic cells are found in the liver of GSN knockout mice^52^. In our previous study, we performed RNA interference in PC12 cells to examine the effects of cGSN in reducing apoptosis^22^. The results show that after interrupting the expression of cGSN more apoptosis occurred in the PC12 cells, and this was confirmed in this study using Hoechst 33258 staining. All of the studies mentioned above suggest that the expression of cGSN might have a protective effect against apoptotic effects.

Vitamin D is well known as a calcium homeostasis modulator; however, in recent years, the therapeutic effect of vitamin D on MS has attracted more and more attention. A large study published in 2015, which involved genetic data from almost 38,000 people, found that those who had genes that affected their level of vitamin D had a greater risk of developing MS^53^. Experiments in animal models found that mice have an increased susceptibility to EAE when they were fed a vitamin D-deficient diet, while administration of 1,25-(OH)_2_D_3_ in mice could slow the progression of EAE^54^. Our research in rats also achieved the same results^22, 39^. This suggests that vitamin D intake could remit the process of EAE, but cannot prevent it. In our study, an unexpected result that was also obtained was that vitamin D could cause a reduction in the expression of cGSN and pGSN. Considering the protective effect of GSN in EAE, we hypothesized that vitamin D_3_, together with a proper increase of GSN *in vivo* may have a more beneficial effect on treating MS. For the first time we injected LV-GSN to get a higher GSN level in EAE rats during vitamin D_3_ supplementation, and we observed better symptoms and a slowed down progression in EAE as shown in the results.

In order to examine the pathway between vitamin D and GSN, we performed VDR gene interruption and overexpression in PC12 cells in this study and the results showed that the expression of GSN was significantly changed contrary to the level of VDR. VDR is a nuclear transcription factor that can regulate target gene transcription by binding to DNA sequences. At present, 4 kinds of VDR ChIP-seq data from i) THP-1 monocyte-like cells^43^; ii) LS180 colorectal cancer cells^55^; iii) GM10855 and GM10861 lymphoblastoid cells^45^; and iv) LX2 hepatic stellate cells^44^ are available. Research on these have individually reported 1,600–6,200 VDR-specific binding sites that were significantly enriched near autoimmune- and cancer-associated genes. Notable genes with VDR binding included PTPN2 associated with Crohn’s disease and T1D, and IRF8, which was associated with MS^45^. However, the function of VDR in EAE is critical and there is a report that demonstrated that EAE development was markedly suppressed in mice lacking the vitamin D receptor^56^. This indicated that VDR might have a two-sided effect in the EAE process. A reanalyzing of the public VDR ChIP-seq datasets found that there are a total of 23,409 non-overlapping VDR binding sites, of which 75% are unique within the analyzed cellular models; in the meantime, only the minority of genome-wide VDR binding sites contains a DR3-type sequence (VDR preferentially binding sequence)^57^. This indicates that genes regulated by VDR are quite different due to their cell type. Although GSN is not one of the genes reported by the VDR ChIP-seq data, the results of the microarray analysis of 1,25(OH)_2_D_3_-stimulated THP-1 cells show that the expression of GSN could be regulated by VDR^43^. Taking all of the results mentioned above into consideration, we concluded that VDR could regulate the expression of GSN, although further research is needed to confirm whether the regulation is direct.

In summary, our work describes better symptom reduction and a slowed down progression in EAE after the injection of GSN-overexpressed lentivirus into the lateral ventricle and caudal vein, and the beneficial effect of vitamin D or GSN was the best when combined with vitamin D treatment with a proper increase of GSN in EAE. Furthermore, we discovered that the mediation of vitamin D on GSN might occur through VDR on the transcriptional level. This finding suggest that increasing the GSN level or blocking the pathway of VDR-GSN during vitamin D treatment on EAE/MS might enhance the therapeutic effect of vitamin D.

## Methods

### Collection and Preparation of CSF and Blood Specimens

The CSF and blood we used in this study were collected at Qilu Hospital of Shandong University (Jinan, Shandong Province, China), from MS patients and patients with other neurological disorders (ONDs). CSF was collected by a lumbar puncture which was performed in the L4–L5 intervertebral space of the patients by the doctor for regular diagnose, and only samples that were brilliantly clear without blood or any other impurities were used in the research. Subsequently CSF and blood samples (2 ml) were centrifuged for 10 min at 16,000 × g (4 °C). Then the supernatant was transferred to a new Eppendorf tube and stored at −80 °C until further experimentation. Patients who participated in the study were provided written informed consent and diagnosed according to the criteria of McDonald^58^. The CSF we used in this study was the remaining of the disease diagnose and the way of CSF collection does no harm to the patients. All experiments were approved by the Ethics Committee of School of Medicine, Shandong University, and conducted in accordance with the ethical guidelines of the Declaration of Helsinki of the World Medical Association.

### Animals and EAE Induction

We purchased 6–7-week-old Male Lewis rats from Vital River (Beijing, China). Rats were raised 1 week under standard laboratory conditions with access to standard diet (Laboratory Animal Center of Shandong University, Jinan, China) and clean water before EAE induction. All experimental procedures involving the animals were previously approved by the Animal Care Committee of Shandong University. The experiments were performed in accordance with the US National Institutes of Health Guide for the Care and Use of Laboratory Animals and conformed to the ARRIVE guidelines. The 7–8-week-old male Lewis rats were immunized with MBP (M2295, Sigma-Aldrich, St. Louis, MO, USA) solution dissolved in saline (1 mg/mL) and Bacillus Calmette-Guerin (5 mg/mL, National Vaccine and Serum Institute, Beijing, China) was added in to complete Freund’s adjuvant (F5506, Sigma-Aldrich) to induce EAE. The details of EAE induction and scoring performed in the study was done according to previous methods^39^. Three pairs of EAE and comparable rats were weighed and scored until Day 25 to observe the whole progress of disease. Other rats were euthanized on Day 12 and used in the subsequent experiments. The change of weight and scores for whole progress of disease are showed in Supplementary Fig. S2.

### Injection of GSN Overexpression Lentivirus and Vitamin D Supplement

We first divided 48 Lewis rats into 2 groups due to their EAE induction or not: the control group (C) and the acute EAE group (E). Then each group was separated into 4 groups based on whether or not they were injected with LV-GSN (Genechem, Shanghai, China) and supplied with vitamin D individually or together. The details of the groups are presented in Fig. 1.

According to our prior research^39^, the time of EAE onset was about 8 days after being immunized with MBP, so we performed the LV-GSN injection 3 days after the immunization considering it takes 5 days for the LV-GSN to increase the GSN concentration. For brain injection of the virus, rats were positioned in a stereotaxic instrument after being anesthetized with 5% chloralhydrate (0.6 ml/100 g) via intraperitoneal injection, then LV-GSN (2*10E7 TU per each rat) was injected into the lateral ventricle (AP −1.0 mm, ML −1.5 mm, DV −4.5 mm according to the rat brain in stereotaxic coordinates^59^) at a rate of 3 μl/min. The syringe was left in place for 20 min before being slowly withdrawn from the brain. After being injected into the lateral ventricle, LV-GSN (5*10E7 TU per each rat) was administered intravenously into rats. The dosage of the LV-GSN used in the study was based on the recommendation of Genechem Company. The method of vitamin D_3_ supplementation was carried out as described^60^ with 3 μg/day vitamin D_3_ added to the diet for each rat. Fresh food was provided to rats every other day for 2 weeks prior to euthanasia.

### Histopathology

Spinal cord paraffin sections from rats were stained with haematoxylin and eosin (H&E) to evaluate inflammatory infiltration, based on our previous study^39^. After HE staining, the slides were observed under bright field microscopy (Nikon, Tokyo, Japan). The percentage of lesions was calculated according to previous methods^60^. Briefly speaking, we divided the meninges, white matter, and gray matter of the spinal cord into different quadrants, and each region was scored as 0, 1, 2, and 3, based on the degree of inflammatory cell infiltration in a blinded fashion. The score was recorded as the percentage of lesion in the spinal cord. Each spinal cord section was divided into quadrants; 15 quadrants/slide and 2 slides/mouse were scored.

### Analysis of 25-(OH) D_3_ and 1,25-(OH)_2_D_3_

Blood was obtained directly from the heart, after centrifuging for 10 min at 16,000 × g (4 °C), serum was transferred into a new Eppendorf tube, and the concentrations of 25-hydroxyvitamin D_3_ (25-(OH) D_3_) and 1, 25-dihydroxyvitamin D_3_(1, 25-(OH)_2_D_3_) were determined in duplicate with enzyme-linked immunosorbent assay (ELISA) kits (CUSABIO, Wuhan, Hubei, China) according to the manufacturer’s instructions.

### Real-time PCR

Total RNA was extracted from the spinal cord (30 mg) or PC12 cells using the RNeasy Purification Kit (Qiagen, Hilden, Germany), according to the manufacturer’s protocols. Micro-spectrophotometer (Thermo, Carlsbad, CA) was used to measure the quantity and purity of RNA. Reverse transcription was performed to synthesize first-strand cDNA using EasyScript One-Step gDNA Removal and cDNA Synthesis SuperMix (TRANS), and SYBR Green PCR Master Mix (Roche, Germany) was used to achieve amplification. The reaction was performed in the CFX96 Real-Time PCR System (Bio-Rad). Each sample was run in triplicate. The procedures carried out in reverse transcription and real-time PCR were all based on the manufacturer’s protocols. GAPDH was used as housekeeping mRNA to normalize transcript VDR or GSN abundance. The relative concentration of VDR or GSN mRNA was analyzed using the x = 2^−∆∆CT^ method.

### Western Blotting

To assess the GSN expression level in MS, we collected CSF and serum from MS and OND patients. Meanwhile, we also gathered the serum and spinal cords of Lewis rats to investigate the difference in protein expression between the different groups. Tissue samples were lysed in RIPA Lysis Buffer (Beyotime, Shenzhen, Guangdong, China) and soon afterwards homogenized with a tissue homogenizer on ice and centrifuged (10 min, 16,000 × g, 4 °C). Then the supernatants from tissue homogenates (500 μl), CSF samples (500 μl), and serum (10 μl) were precipitated with 100% ice-cold acetone and stored overnight at −20 °C. On the next day, pellets were solubilized in RIPA lysis buffer after centrifugation. Protein concentrations were detected by BCA protein assay kit (Beyotime). We separated 20 μg of protein of each sample by 10% SDS-PAGE and transferred them to PVDF membranes (Millipore, Billerica, MA, USA). After blocking in 5% non-fat dry milk, which was dissolved in TBS-T at room temperature for 1.5 h, PVDF membranes were incubated with GSN primary antibody (1:1000) or anti-VDR antibody overnight at 4 °C. Then, membranes were washed with TBS-T 3 times and incubated with horseradish peroxidase-labeled anti-rabbit IgG (1:5000, ZSGB-BIO, Beijing, China). Finally, the immunoreactive complexes were visualized by the Lumina Enhanced Chemiluminescent Kit (Millipore, Billerica, MA, USA) and the light was detected with photographic film. Actin quantification was used as an internal standard to correct for variations in total protein loading.

### Cell Culture

The PC12 cell used in this study was purchased from ATCC: it is a sympathetic nerve cell line derived from rat pheochromocytoma^61^. Cells were cultured in complete medium containing 90% (v/v) Dulbecco’s modified Eagle’s medium (DMEM) (Hyclone), 10% (v/v) fetal bovine serum (Gibco), and 1% (v/v) penicillin and streptomycin in 75 cm^2^ cell culture flasks. Cell growth was maintained in a humidified atmosphere containing 5% CO_2_ at 37 °C.

### RNA Expression

GSN expression lentivirus vector (LV-GSN-EGFP) or VDR expression plasmid (VDR-EGFP, GV230, Genechem, Shanghai, China) was transfected into PC12 cells to facilitate the stable expression of GN/VDR. Alternatively, a control lentivirus vector or plasmid (NC) was injected into PC12 cells. Transfections were performed when PC12 cells reached 70–80% confluence in 6-well plates. VDR expression plasmid and its control plasmid were transfected using Lipofectamine 2000 transfection reagent (Invitrogen, Carlsbad, USA) according to the manufacturer’s instructions. The efficiency of GSN or VDR transfections was confirmed by observing the fluorescence expressed by EGFP under blue ray field by microscopy (Nikon, Tokyo, Japan), and the protein level was verified by Western blotting and RT-PCR was also carried out on the RNA level.

### RNA Interference

A set of 19-mer shRNA oligos (target sequence: GGAAGTACAGGGAGCTATT) were designed and synthesized (VDR-RNAi, Genechem, Shanghai, China) to interfere with VDR expression. A lentiviral expression vector (GV118, Genechem, Shanghai, China) and control lentiviral expression vector were used to ensure transfection efficiency. We also designed and synthesized a set of 21-mer siRNA oligos (siGSN; GenePharma, Shanghai, China) to interfere with gelsolin expression; the target sequence is CGGTGACTGCTTCATTCTGTT. A control siRNA (NC) was also used. The PC12 cells used in this experiment were grown in 6-well plates and transfected at 30–50% confluence. Transfections were performed according to the manufacturer’s instructions. Before siGSN transfection, the medium was changed to serum-free medium and Lipofectamine 2000 transfection reagent (Invitrogen, Carlsbad, USA) was used in GSN interference to ensure transfection efficiency. The reduction of gelsolin and VDR expression by siRNA was verified by Western blotting and RT-PCR.

### Nuclear Staining with Hoechst 33258

The PC12 cells were seeded into 24-well plates at 40% confluency and transfected with either LV-GSN, siGSN, NC lentiviral or NC siRNA using the transfection agent at room temperature for 24 h and then they were treated with 20 ng/ml rat TNF-α (Peprotech, USA). Apoptotic cells were detected at 48 and 72 h after being treated with rat TNF-α using Hoechst 33258 (Beyotime Institute of Biotechnology, Shanghai, China), according to the manufacturer’s protocol. A fluorescent microscope (Eclipse TE2000-U; Nikon Corp., Tokyo, Japan) was used to capture images of the stained cells under at 350 nm excitation and 460 nm emission.

### Data analysis

Individual mice were analyzed, and the mean and SD were calculated for each group of mice. The group sizes are given in the Fig. 1 and figure legends. The significance of differences between the group means was determined using the one-way analysis of variance (abbreviated one-way ANOVA) and Student’s *t* test, as indicated^62^. A value of *p* < 0.05 was considered significant.

## Electronic supplementary material

Supplementary Information
