# Supplementary material for: Overexpression of GSN could decrease inflammation and apoptosis in EAE and may enhance vitamin D therapy on EAE/MS
Source: Sci Rep. 2017 Apr 4;7:604. doi: 10.1038/s41598-017-00684-w (PMC5428824; doi:10.1038/s41598-017-00684-w)

Overexpression of GSN could decrease inflammation and apoptosis in EAE and may enhance vitamin D therapy on EAE/MS

Jifang Gao, Zhaoyu Qin, Xinyuan Guan, Juanjuan Guo, Huaqing Wang, Shilian Liu

Fig. S1 A, The uncropped full-length gels of Fig.5B-I. B, The uncropped full-length gels of Fig. 6A-I and B-I. C, The uncropped full-length gels of Fig. 2G-I. D, The uncropped full-length gels of Fig. 2H-I.

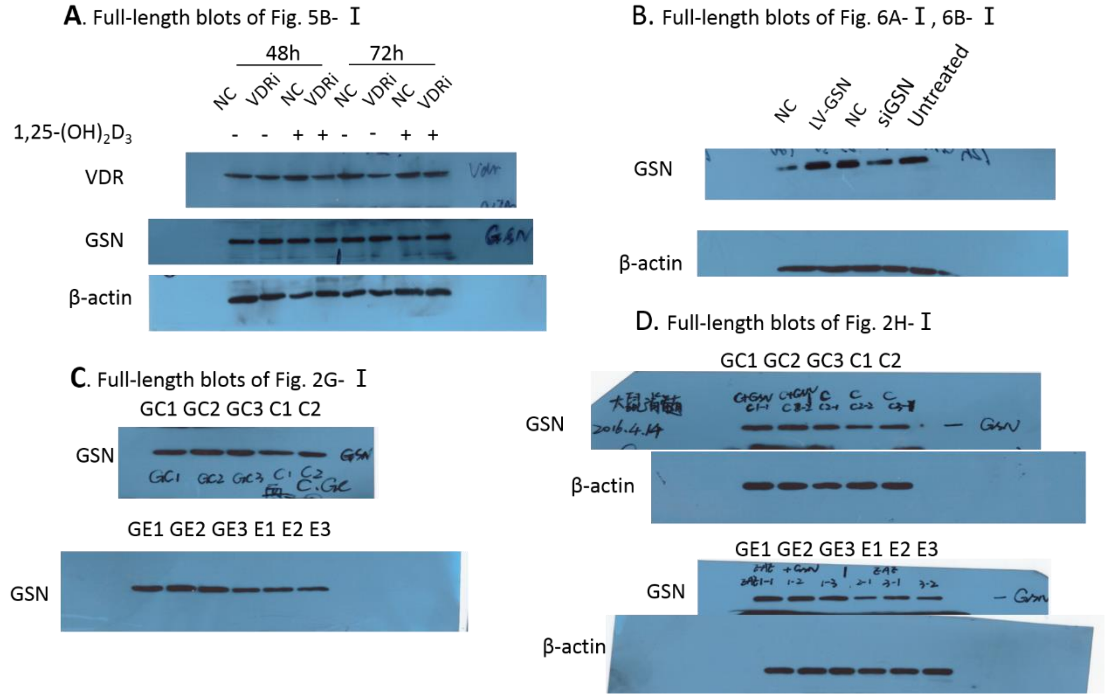

Fig. S2 A, The weight changes of EAE rats during the whole progress of EAE. B, Scores of the EAE rats during the whole progress of EAE.

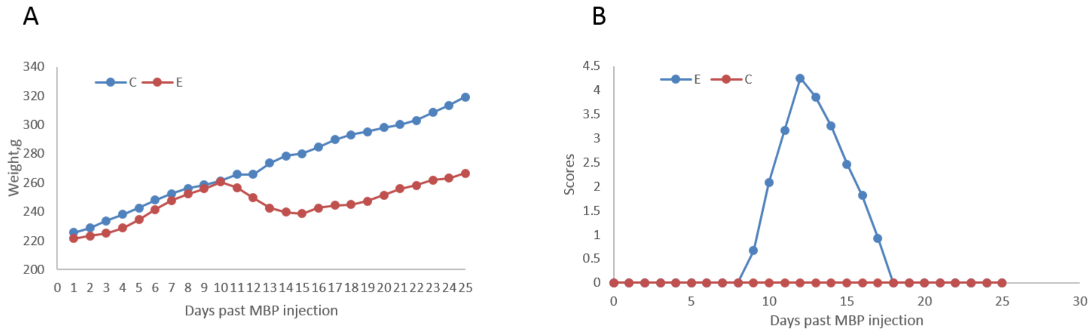

Supplement: Supplementary file 1 — Supplementary Information [file 41598_2017_684_MOESM1_ESM.pdf]
